# Effectiveness and safety of a prospective audit and feedback-based antimicrobial stewardship program in hospitalized COVID-19 patients: a quasi-experimental before-and-after study

**DOI:** 10.3389/fphar.2026.1662195

**Published:** 2026-03-20

**Authors:** Ariana Martínez-Suárez, Elena Salamanca-Rivera, Jaime Cordero-Ramos, Jesús Rodríguez-Baño, Pilar Retamar-Gentil

**Affiliations:** 1 Pharmacy Department, University Hospital Virgen Macarena, Seville, Spain; 2 Pharmacy Department, University Hospital Son Espases, Palma de Mallorca, Spain; 3 Unidad Clínica de Enfermedades Infecciosas y Microbiología, Instituto de Biomedicina de Sevilla (IBiS), Hospital Universitario Virgen Macarena, CSIC, Seville, Spain; 4 Centro de Investigación Biomédica en Red en Enfermedades Infecciosas (CIBERINFEC), Madrid, Spain; 5 Departamento de Medicina, Universidad de Sevilla, Sevilla, Spain

**Keywords:** antibiotics, antimicrobial stewardship, feedback, prospective audit, SARS-CoV-2

## Abstract

**Background:**

Antibiotic use among hospitalized patients with confirmed active SARS-CoV-2 infection is notably high (>70%) compared to the incidence of bacterial coinfections and superinfections (3.5% and 14.3%, respectively). Unjustified antimicrobial use poses preventable risks due to its toxicity and potential for long-term negative consequences. In this context, antimicrobial stewardship programs (ASPs) play a pivotal role in optimizing antibiotic therapy for COVID-19 patients.

**Methods:**

A quasi-experimental, before-and-after study was conducted to assess the impact of the COVID-ASP. The pre-ASP cohort included patients retrospectively, while the ASP cohort included patients prospectively, following the program implementation. The primary outcome was the evaluation of the impact of the COVID-ASP on days of therapy (DOT)/1,000 patient-days. Secondary outcomes included the rate of antibiotic use, rate of inappropriate use, number of recommendations made, their acceptance rate, the incidence of *Clostridioides difficile* infection, 30-day mortality, and 30-day readmissions.

**Results:**

A total of 1,289 patients admitted were included, 561 (43.5%) in the pre-ASP cohort and 728 (56.5%) in the ASP cohort. The COVID-ASP cohort showed a significantly lower DOT/ 1,000 patient-days (162.96 vs. 105.71; p < 0.001). Additionally, the COVID-ASP was associated with a significant reduction in the rate of antibiotic use for suspected pneumonic coinfections (13.2% vs. 5.9%, p < 0.001), for all causes (17.5% vs. 12.5%, p = 0.012), and for pneumonic superinfection (9.3% vs. 5.9%, p = 0.022). No significant difference was observed in antibiotic use for superinfection from all causes (16.0% vs. 16.2%, p = 0.936). The program also reduced the inappropriate antibiotic use rate for suspected pneumonic coinfection (8.7% vs. 1.9%, p < 0.001), for all causes (10.3% vs. 4.0%, p < 0.001), and for pneumonic superinfection (4.3% vs. 1.9%, p = 0.013). However, it showed no impact on inappropriate antibiotic use in superinfections across all causes (5.9% vs. 5.2%, p = 0.605). The most common recommendations included discontinuing antibiotics (58.6%) and adjusting the antibiotic regimen (30.3%). The program showed no significant effect on the incidence of *Clostridioides difficile* infections (0% vs. 0.1%, p = 0.379), 30-day mortality (15.2% vs. 18.1%, p = 0.156), or 30-day readmission rates (4.8% vs. 4.9%, p = 0.469).

**Conclusion:**

In our experience, the three-step evaluation methodology allows for the evaluation of antibiotic prescriptions in patients with COVID-19 and allows for their optimization.

## Introduction

The COVID-19 pandemic, caused by the SARS-CoV-2, exposed the vulnerability of global healthcare systems, compelling governments and institutions to coordinate efforts to respond efficiently and collaboratively to an unprecedented challenge.

The pandemic has been associated with the indiscriminate use of antibiotics, which, according to some authors, may mark the beginning of the “post-antibiotic era” (Kwon and Powderly, 2021; Owoicho et al., 2021). COVID-19 management guidelines recommend initiating antibiotic treatment only when there is a well-founded clinical suspicion of additional bacterial coinfection or superinfection (Sturza et al., 2023). Despite the recommendations, 74.6% of patients admitted to the hospital received antibiotics (Langford et al., 2021). In contrast, a meta-analysis determined that the incidence of bacterial coinfection and superinfection was 3.5% (95% CI, 0.4–6.7) and 14.3% (95% CI, 9.6–18.9) of patients, respectively (Langford et al., 2020). The similarity in symptomatology, as well as in laboratory results and radiographic findings, to community-acquired bacterial pneumonia, the uncertainty surrounding the pandemic, and the absence of treatments with proven efficacy are factors that may have contributed to the excessive prescription of antibiotics in COVID-19 patients. The unjustified use of antimicrobials introduces an avoidable risk of potential adverse outcomes due to the high toxicity potential and long-term negative effects associated with antibiotics (Spernovasilis and Kofteridis, 2021).

Antimicrobial stewardship programs (ASPs) based on non-taxation audits and feedback are designed to provide specific guidance regarding prescribed antibiotics that are considered amenable to optimization (Dellit et al., 2007; Rodríguez-Baño et al., 2012). During the pandemic, many hospital resources were diverted to the care of COVID-19 patients, affecting the usual activities, including those related to ASP (Ashiru-Oredope et al., 2021; Wimmer et al., 2022; Leung et al., 2022). Since the onset of the pandemic, there has been a call to implement high-value ASP to contribute to the identification, selection, and treatment of patients affected by COVID-19 (Huttner et al., 2020; Mazdeyasna et al., 2020; Stevens et al., 2020). Some experiences have been published as the COVASP clinical trial that demonstrates that prospective audit and feedback are effective and safe in optimizing and reducing antibiotic use in adults admitted to hospital with COVID-19 (Chen et al., 2023). We present the results of implementing a specific three-step antimicrobial stewardship program for hospitalized patients with a confirmed diagnosis of COVID-19 (COVID-ASP), based on a pharmacist pre-evaluation followed by prospective audit and feedback.

## Materials and methods

### Study design and setting

A quasi-experimental before-and-after study was conducted to evaluate a prospective audit-and-feedback intervention as part of an antimicrobial stewardship program for patients admitted with SARS-CoV-2 infection (COVID-ASP) at the Virgen Macarena University Hospital (HUVM).

The Virgen Macarena University Hospital is a third-level hospital of the Andalusian Public Health System. It has a 900-bed capacity and provides healthcare to the northern area of the city and the province of Seville, with a population of close to half a million inhabitants. The hospital has been actively promoting the appropriate use of antimicrobials since 1997, including educational activities, elaboration of local guidelines, measurement of antibiotic consumption, and a follow-up program for bacteremia patients. The hospital’s ASP was officially established in 2013.

The pre-intervention cohort (before) was performed during the first period of the COVID-19 pandemic (March–December 2020), and the intervention cohort (after) prospectively included patients during the implementation of an antimicrobial stewardship program in COVID-19 patients (January–June 2021).

### Participants

Adult patients with a diagnosis of active SARS-CoV-2 infection confirmed by RT-PCR or antigen detection from nasal and/or oropharyngeal swabs, or lower tract respiratory specimens, admitted to the pulmonology, internal medicine, and infectious diseases departments were included. Patients admitted to intensive care units and those with limited therapeutic options were excluded.

### Procedures

Before the implementation of COVID-ASP, patients were primarily managed by COVID-19 physician teams, usually including at least one internal medicine, pulmonology, or infectious diseases physician. During the pre-intervention period, the usual ASP activities were diminished due to work overload caused by the high patient care burden of COVID-19 patients. The prescriptions made during the pre-intervention period were retrospectively evaluated by the COVID-ASP team.

In January 2021, a comprehensive three-phase ASP, based on prospective audit and feedback, was implemented for hospitalized patients with confirmed SARS-CoV-2 infection.

### Audit procedures

The first step, conducted by clinical pharmacists, involved screening and preliminary evaluation of all active antibiotic prescriptions on weekdays. Prescriptions in the Emergency Department and surgical prophylaxis were excluded. The clinical pharmacists performed an initial assessment of the antimicrobial, including dosage, route of administration, and duration. The clinical pharmacist systematically collected a predefined minimum dataset in an MS Excel^®^ spreadsheet, previously agreed upon with the multidisciplinary stewardship team. Data collection was based on information available in the electronic prescription system, electronic medical records, laboratory results, and microbiology reports. The following information was recorded in a structured format: a) Patient history: previous microbiological findings, antimicrobial treatments (agents, doses, and duration) administered during the current admission, prior hospitalizations, and in outpatient settings over the previous year; and risk factors for infections caused by ESBL-producing *Escherichia coli* or *Klebsiella* spp. Additional risk factors for *Enterococcus* spp. infections were also collected. b) Current infection (clinical, laboratory, and microbiology): clinical rationale for initiating antibiotic therapy; relevant laboratory parameters and their evolution in recent days; available microbiological results; radiological findings from chest X-rays or other imaging studies, and vital signs. c) Characteristics of the current antimicrobial therapy: antibiotic agents, doses, and route of administration. Using this information, the clinical pharmacist performed an initial assessment of the antimicrobial regimen, including evaluation of the indication, dose, route of administration, expected duration, and adherence to the local antibiotic guideline. The second step involved discussion of all inappropriate prescriptions between the clinical pharmacist and the infectious disease physician. If the prescription could be optimized, the infectious disease physician made a written, non-mandatory recommendation to the attending physician. Direct contact between the prescribing physician and the infectious diseases specialist was not always possible. The recommendations included suggestions for diagnostic tests, discontinuation of treatment, drug change, and/or adjustments in duration, dose, or route of antibiotic administration. In the third step, the pharmacist checked whether the recommendations made during the second phase were accepted. Recommendations were considered accepted if the proposed changes were implemented within 24 h of the recommendation. If the recommendation was not accepted, the patient would be re-audited on the next weekday. The retrospective review was performed by the same pharmacist involved in the prospective assessments, and in cases of uncertainty, the pharmacist consulted with the infectious diseases specialists to ensure consistency in the evaluation.

### Outcomes and definitions

The study measured process and clinical outcomes. The primary outcome was to evaluate the impact of the COVID-ASP on days of therapy (DOT) per 1,000 patient-days. Secondary outcomes included determining the rate of antibiotic use and inappropriate use in cases of coinfection and superinfection, 30-day readmission rates following hospital discharge, 30-day mortality post-admission, and *Clostridioides difficile* infection (CDI) 3 months post-admission. We also assessed the recommendations made by the COVID-ASP and the acceptance rate by physicians. The antibiotic use rate is defined as the number of patients receiving antibiotics relative to the total number of hospitalized patients.

Coinfection is defined as the suspicion of a bacterial infection not explained by the clinical presentation caused by the SARS-CoV-2 virus, or a confirmed infection that becomes evident within the first 48 h following the confirmation of active SARS-CoV-2 infection.

A patient is considered to have a superinfection when a bacterial infection is suspected that is not explained by the clinical presentation caused by the SARS-CoV-2 virus or when a confirmed infection becomes evident after the first 48 h following confirmation of the active infection.

### Statistical analysis

The mean and standard deviation or median and interquartile range, as appropriate, were used to average the quantitative variables. Differences in DOT/1,000 patient-days were determined using the Poisson test. Differences between quantitative variables were measured using Student’s t-test or the Mann–Whitney U test. To analyze differences between qualitative variables, the chi-squared or Fisher test was used. To study the relationship between quantitative variables and dichotomous qualitative variables, the Mann–Whitney U test was used. Multivariate analyses were performed using binary logistic regression and Cox regression, as appropriate. Variables with a p-value <0.20 in bivariate analyses and variables of clinical relevance were included in the initial multivariable models. Backward stepwise elimination was used to derive the final models. Collinearity was assessed using variance inflation factors. Model stability was evaluated considering the number of events per variable. Sensitivity analyses were performed stratified by age (<65 vs. ≥ 65 years) and by FEN-COVID phenotype, a composite classification integrating baseline severity, clinical presentation, and laboratory parameters. Analyses were performed using SPSS version 25 software.

### Ethical

The project was approved by the Ethics Committee of the Virgen Macarena and Virgen del Rocío University Hospitals (internal code 2848-N-21).

### Source of financing

There is no funding source for this project.

## Results

### Study population

In all, 1,289 patients were included, 561 (43.5%) in the pre-ASP cohort (retrospective audit) and 728 (56.5%) in the ASP cohort (prospective audit and feedback). Demographic, baseline, admission, and COVID-19 therapy characteristics of patients are shown in Table 1. Patients in the pre-ASP cohort were significantly younger (64 years vs. 67 years). Hematologic malignancies were more commonly observed in patients from the ASP cohort. Cough and diarrhea were more common in the pre-ASP cohort. Baseline oxygen saturation without supplemental oxygen, lymphocyte, and platelet counts were higher in the pre-ASP cohort, while C-reactive protein (CRP) levels were higher in the ASP cohort. Patients in the ASP cohort exhibited a higher degree of severity (measured using the FEN-COVID scale). In the ASP cohort, a higher proportion of patients received COVID-19-specific therapies. In the pre-ASP cohort, the incidence of bacterial coinfection was 1.8%, while it was 2.3% in the intervention cohort. The incidence of superinfection was 4.2% and 4.0%, respectively. No statistically significant differences in the incidence of bacterial infections associated with COVID-19 were observed in both cohorts. Blood cultures yielded bacterial growth in 32 (2.5%) of 1,289 patients, and urine cultures yielded bacterial growth in 34 (2.6%) patients.

**TABLE 1:** Demographic, baseline, admission, and COVID-19 therapy characteristics of patients included in the pre-ASP and ASP cohorts.

Variables	Pre-ASP (n = 561)	ASP (n = 728)	p-value
Demographic characteristics			
Age (mean, IQR), years	64.0 (49.0–76.0)	67.0 (53.0–78.0)	0.014
Sex (male), n (%)	329 (58.6)	442 (58.0)	0.807
Baseline characteristics			
Underlying disease, n (%)			
Chronic lung disease	85 (15.2)	131 (18.0)	0.175
Obesity	94 (16.8)	147 (20.2)	0.117
Chronic heart disease	167 (29.8)	196 (26.9)	0.260
Arterial hypertension	286 (51)	391 (53.7)	0.331
Asthma	42 (7.5)	57 (7.8)	0.819
Chronic kidney disease	37 (6.6)	52 (7.1)	0.701
Cirrhosis	6 (1.1)	8 (1.1)	0.960
Active solid cancer	29 (5.2)	56 (7.7)	0.070
Hematologic malignancy	4 (0.7)	15 (2.1)	0.047
Diabetes mellitus	128 (22.8)	192 (26.4)	0.143
Dementia	35 (6.2)	53 (7.3)	0.462
Basal treatment, n (%)			
Chemotherapy	6 (1.1)	10 (1.4)	0.625
Biological treatment	2 (0.4)	3 (0.4)	0.874
Admission characteristics			
Symptoms, n (%)			
Fever	382 (68.1)	497 (68.3)	0.946
Dyspnea	327 (58.3)	454 (62.4)	0.138
Cough	383 (68.3)	428 (58.8)	<0.001
Diarrhea	121 (21.6)	113 (15.5)	0.005
Anosmia	34 (6.1)	31 (4.3)	0.143
Clinical parameters			
Temperature (mean, IQR), °C	36.7 (36–37.5)	36.5 (36–37.4)	0.004
Diastolic blood pressure (mean, IQR), mmHg	78 (70–87)	76 (69–87)	0.497
Systolic blood pressure (mean, IQR), mmHg	127 (115–140)	125 (111–141)	0.426
Saturation basal (mean, IQR), %	94 (91–97)	92 (89–94)	<0.001
Analytical parameters			
Hemoglobin (mean, IQR), g/dL	13.9 (12.7–15)	13.8 (12.5–15.1)	0.612
Leukocytes (mean, IQR),/μL^3^	6,350 (4,835–8,550)	6,250 (4,840–8,557.5)	0.802
Neutrophils (mean, IQR),/μL^3^	4,670 (3,270–6,550)	4,640 (3,370–6,962.5)	0.438
Lymphocytes (mean, IQR),/μL^3^	1,060 (800–1,465)	900 (650–1,290)	<0.001
Platelets (mean, IQR),/μL^3^	201 (145–268)	183 (93.3–250)	<0.001
Creatinine (mean, IQR), mg/dL	0.92 (0.76–1.15)	0.89 (0.72–1.13)	0.058
CRP (mean, IQR), mg/L	60.2 (28.9–121.7)	79.2 (40.4–145.9)	<0.001
PCT (mean, IQR), ng/mL	0.09 (0.06–0.22)	0.11 (0.06–0.24)	0.058
IL-6 (mean, IQR), pg/mL	23.65 (8.88–51.95)	24.5 (7.56–59.7)	0.785
DD (mean, IQR), ng/mL	669.0 (425.0–1,138.0)	696 (436–1,200.5)	0.564
COVID-19 phenotype, n (%)			
A	104 (18.5)	30 (4.1)	<0.001
B	437 (77.9)	650 (89.3)	<0.001
C	20 (3.6)	48 (6.6)	0.016
COVID-19 therapy, n (%)			
Remdesivir	17 (3)	47 (6.5)	0.005
Corticosteroids	406 (72.4)	681 (93.5)	<0.001
Tocilizumab	27 (4.8)	125 (17.2)	<0.001

Antibiotic prescriptions for coinfection in the pre-ASP and ASP cohorts were due to the following sources: pneumonia (13.2% vs. 5.9%), urinary tract infection (3.5% vs. 3.3%), non-pneumonic respiratory infection (0.5% vs. 1.4%), and others (1.4% vs. 1.8%). The most frequently prescribed antibiotics were ceftriaxone (11.4% vs. 6.0%), followed by amoxicillin/clavulanic acid (2.0% vs. 2.1%) and piperacillin/tazobactam (1.8% vs. 1.8%). Antibiotic prescriptions for superinfection were due to the following sources: pneumonia (9.3% vs. 5.9%), urinary tract infection (3.0% vs. 4.5%), primary bacteremia (1.1% vs. 1.1%), non-pneumonic respiratory infection (1.1% vs. 0.7%), and others (1.7% vs. 3.9%). The most frequently prescribed antibiotics for superinfection were ceftriaxone (5.9% vs. 4.5%), piperacillin/tazobactam (4.5% vs. 4.9%), and amoxicillin/clavulanic acid (1.6% vs. 1.1%).

The results for the primary outcome are detailed in Table 2. The COVID-ASP cohort showed a significantly lower DOT/1,000 patient-days (105.71 vs. 162.96; rate ratio (RR), 0.65; 95% CI, 0.58–0.76; p < 0.001).

**TABLE 2:** Primary and secondary outcomes.

Variables	Pre-ASP (n = 561)	ASP (n = 728)	OR (95% CI)	p-value
Primary outcome				
DOT/1,000 patient-days	162.96	105.71	0.65 (0.58–0.76) ^a^	<0.001
Secondary outcomes				
Antibiotics for bacterial pneumonia coinfection^b^, n (%)	74 (13.2)	43 (5.9)	0.41 (0.28–0.61)	<0.001
Antibiotics for bacterial coinfection^b^ (all causes), n (%)	98 (17.5)	91 (12.5)	0.68 (0.50–0.92)	0.012
Antibiotics for bacterial pneumonia superinfection^c^, n (%)	52 (9.3)	43 (5.9)	0.61 (0.40–0.94)	0.022
Antibiotics for bacterial superinfection^c^ (all causes), n (%)	90 (16)	118 (16.2)	1.01 (0.75–1.37)	0.936
Incorrect use of antibiotics for bacterial pneumonia coinfection^b^, n (%)	49 (8.7)	14 (1.9)	0.20 (0.11–0.37)	<0.001
Incorrect use of antibiotics for bacterial coinfection^b^ (all causes), n (%)	58 (10.3)	29 (4.0)	0.36 (0.22–0.57)	<0.001
Incorrect use of antibiotics for bacterial pneumonia superinfection^c^, n (%)	24 (4.3)	14 (1.9)	0.44 (0.22–0.86)	0.013
Incorrect use of antibiotics for bacterial superinfection^c^ (all causes), n (%)	33 (5.9)	38 (5.2)	0.88 (0.55–1.42)	0.605
CDI	0 (0.0)	1 (0.1)	-	0.379
Mortality	85 (15.2)	132 (18.1)	1.22 (0.93–1.60)^d^	0.153
All-cause readmissions	27 (4.8)	29 (4.0)	0.82 (0.48–1.40)	0.469

^a^ Incidence rate ratio (RR).

^b^ Coinfection was defined as either a suspected bacterial infection not explained by the symptoms caused by SARS-CoV-2, or a confirmed infection, identified within the first 48 h after confirmation of active SARS-CoV-2 infection.

^c^ Superinfection was considered when either a suspected bacterial infection not explained by the symptoms caused by SARS-CoV-2, or a confirmed infection, was identified after the first 48 h following the confirmation of active infection.

^d^ Hazard ratio (HR).

Regarding secondary outcomes, the intervention was associated with a reduction in the rate of antibiotic use for suspected pneumonic coinfection (13.2% vs. 5.9%; p < 0.001) and for all causes (17.5% vs. 12.5%; p = 0.012). Additionally, the rate of antibiotic use for suspected pneumonic superinfection was lower in the intervention cohort (9.3% vs. 5.9%; p = 0.022).

Factors independently associated with a prescription of antibiotics for coinfection were chronic lung disease, solid cancer, dementia, baseline treatment with biologics, leukocytes at admission ≥14,000/μL^3^, lymphocytes at admission <1,000/μL^3^, and CRP at admission ≥100 mg/L. The ASP period was independently associated with a lower prescription of antibiotics for coinfection (Supplementary Table S1). Variables independently associated with prescription of antibiotics for superinfection were age ≥65 years, chronic lung disease, chronic kidney disease, active solid cancer, dementia, treatment with systemic corticosteroids during hospitalization, and treatment with tocilizumab during hospitalization (Supplementary Table S2). Factors independently associated with inappropriate antibiotic prescription for coinfection were active solid cancer, CRP ≥100 mg/L, and baseline oxygen saturation <92%. The ASP period was independently associated with a lower rate of antibiotic prescription (Supplementary Table S3). Variables independently associated with inappropriate antibiotic prescription for superinfection were age ≥60 years, obesity, active solid cancer, dementia, baseline corticosteroid treatment, and tocilizumab treatment during hospitalization (Supplementary Table S4).

The COVID-ASP intervention showed no effect on 30-day readmission rates post-discharge or on mortality within 30 days following admission. Additionally, in the multivariate analysis, COVID-ASP showed no impact on 30-day mortality (HR, 0.87; 95% CI, 0.66–1.16; p = 0.346) or on 30-day readmission after hospital discharge (OR, 0.68; 95% CI, 0.38–1.20; p = 0.183). On the other hand, it did not demonstrate an impact on CDI.

All prescriptions were audited every working day without exception. A total of 637 audits and 99 recommendations were made. The reasons for the recommendations were 3 (3.0%) inadequate, 28 (28.3%) adequate but not recommended, 32 (32.3%) unnecessary, 28 (28.3%) inadequate duration, and 8 (8%) inadequate route of administration. The recommendations provided by the COVID-ASP program were as follows: 60 (60.6%) advised discontinuing antibiotic use, 30 (32.3%) recommended modification of the antibiotic, and 8 (8.1%) suggested changing the route of administration. The acceptance rate of the recommendations was 96/99 (97.0%).

## Discussion

A quasi-experimental before-and-after study has been designed in which a COVID-ASP was implemented for hospitalized patients with a confirmed diagnosis of infection caused by the SARS-CoV-2 virus. During the ASP period, all prescriptions were audited by ASP members. Due to the high acceptance rates of the recommendations, nearly all patients received optimal antibiotic therapy. Overall, prospective audit and feedback demonstrated a reduction in DOT/1,000 patient-days (105.71 vs. 162.96; p < 0.001) and in the rate of inappropriate prescriptions (15.3% pre-ASP vs. 8.4% ASP; p < 0.001) without impacting the readmission rate or mortality of the intervened patients. The absence of effect on antibiotic use for superinfections likely reflects that most were nosocomial infections routinely managed through infectious diseases consultation within our long-standing ASP program. As these cases were already optimized before the COVID-ASP, the margin for additional improvement was limited. In contrast, the intervention primarily targeted empirical prescribing in suspected coinfections, where diagnostic uncertainty is greater.

At the start date of this study, January 2021, there was no published experience of audit and feedback conducted in COVID-19 patients. Recently, a pragmatic, cluster-randomized, non-inferiority trial was published, which determined that prospective audit and feedback are effective in optimizing and reducing antibiotic use in adults admitted to hospital with COVID-19. Despite the intervention in the COVASP study, DOT/1,000 patient-days remained higher in the intervention cohort than the non-intervention cohort (384.2 DOT vs. 364.9 DOT per 1,000 patient-days), and 53% of the patients received antibiotics during their hospital stay (Chen et al., 2023). In our study, the DOT/1,000 patient-days rate was substantially lower than that reported in the COVASP study. A published before-and-after study that included 298 patients identified an antibiotic use rate of 61.8% in the pre-ASP period, which decreased to 44.4% following the implementation of the ASP intervention (Anderson et al., 2023). Moreover, the rate of antibiotic use in our study was 29.1% in the pre-ASP cohort, suggesting a narrower scope for improvement compared to findings reported in other studies.

In the ASP cohort of our study, patients were older, there were more cases of hematologic malignancy, and a higher proportion of patients were classified as phenotype B and C according to the FEN-COVID scale. Patients in the intervention cohort had lower baseline oxygen saturation, lower lymphocyte count, and higher CRP levels. These data confirm a poorer prognosis for the ASP cohort, which may contribute to a higher prescription rate of antibiotics despite the presence of an ongoing ASP intervention (Berenguer et al., 2020; Izcovich et al., 2020; Zhao et al., 2020; Gutiérrez-Gutiérrez et al., 2021; Martínez-Lacalzada et al., 2021; Khoury et al., 2022; Abdullah et al., 2023). Suranadi et al. (2022), in a retrospective study, determined that antibiotic therapy was associated with higher mortality. In this sense, the use of an antibiotic may represent a surrogate indicator of severity presentation in patients with COVID-19. Patients in the intervention cohort showed a worse prognosis, as assessed by the FEN-COVID-19 scale, a factor that could contribute to an expected increase in antimicrobial therapy prescriptions compared to the ASP cohort. The worse prognosis of patients in the intervention cohort is likely due to changes in hospitalization criteria during the pandemic. Initially, milder cases were admitted due to uncertainty, but as knowledge about the disease improved, only more severe cases were hospitalized (Lee et al., 2022). COVID-19 vaccination data were not considered in the results as the proportion of vaccinated patients was negligible (0.4% pre-ASP vs. 9.7% ASP). In the intervention cohort in our study, remdesivir, tocilizumab, and corticosteroids were used more extensively due to newly available evidence and improved accessibility to these treatments.

The local guidelines of HUVM recommend the empirical use of ceftriaxone in typical pneumonia with admission criteria. In pneumonic coinfection, most prescriptions in both cohorts consisted of ceftriaxone, followed by amoxicillin/clavulanic, with the prescription of piperacillin/tazobactam being infrequent. Finally, the use of combination therapy and azithromycin was noted as anecdotal. Similarly, in bacterial coinfections from all causes, ceftriaxone was the most commonly prescribed antibiotic. In terms of antibiotic prescriptions for pneumonic superinfection, ceftriaxone stands out as the most prescribed antibiotic in both cohorts, followed by piperacillin/tazobactam. The local guidelines of HUVM also recommend ceftriaxone for empirical treatment of early-onset nosocomial pneumonia. Additionally, they suggest empirical treatment with piperacillin/tazobactam for late-onset pneumonia or severe early-onset pneumonia. Similarly, the most commonly prescribed antibiotic for secondary infection from all causes in the pre-ASP cohort was ceftriaxone, followed by piperacillin/tazobactam. In contrast, in the ASP cohort, piperacillin/tazobactam was the most prescribed, followed by ceftriaxone. The higher use of antipseudomonal coverage in the intervention cohort could be attributed to the greater severity of patients, as assessed by the FEN-COVID scale, the higher number of patients with hematologic malignancies, and the resumption of many hospital activities, such as elective surgeries, during the third and fourth waves of the pandemic.

The intervention reduced the rate of antibiotic prescription for suspected coinfection for all causes, as well as the rate of antibiotic use for suspected pneumonic superinfection. The incidence of bacterial infections associated with COVID-19 showed no significant differences between cohorts (coinfection: 1.8% vs. 2.3%; superinfection: 4.2% vs. 4.0%). Despite these reductions, the overall rate of antibiotic use showed no statistically significant differences between the two cohorts (29.1% vs. 26%; p = 0.216), which may be attributed to the relatively low rate of antibiotic prescription compared to data from published cohorts, leaving little room for improvement. The differences observed in our study in terms of coinfections, superinfections, and the rate of antibiotic use were lower than those reported in other studies. In the meta-analysis by Langford BJ et al., 71.9% of patients received antibiotics during hospitalization, with coinfection and superinfection rates of 3.5% and 14.3%, respectively. Similarly, in the COVASP trial, antibiotic use (53%) was much higher than the rate of bacterial infections associated with COVID-19 (4%) (Langford et al., 2021; Anderson et al., 2023; Chen et al., 2023). Considering that 32.3% of the recommendations in the ASP cohort advised discontinuing antibiotics due to being unnecessary, and given the high acceptance rate, it is likely that the antibiotic use rate would have been lower if measured after the intervention. We have no record of published studies identifying factors associated with the inappropriate use of antibiotics in COVID-19, which could be valuable for guiding ASP activities.

Similarly to findings in other published studies, no significant differences were observed in the incidence of CDI (Anderson et al., 2023; Chen et al., 2023). Studies with a larger number of patients and longer follow-up periods are necessary to determine its effect on this variable. ASPs have been proven to be safe in hospitalized patients, including critically ill adults and pediatric patients (Davey et al., 2017; Lindsay et al., 2019; Chorafa et al., 2023). Similar to the COVASP trial, our study demonstrates that prospective audit and feedback in COVID-19 patients did not impact 30-day mortality or readmission rates.

Compared to the COVASP study, similar recommendations were made. Notably, in our study, 32.3% of the recommendations suggested discontinuing antibiotic therapy due to its being unnecessary, compared to 57.0% in the COVASP study. The acceptance rate of recommendations was 84% in the COVASP study, while in our study, it was as higher at 97%. The high acceptance rate likely reflects our hospital’s long-standing ASP culture and established ID–Pharmacy collaboration. Nevertheless, the intervention relies on simple audit-and-feedback procedures that are easily scalable and do not require substantial resources, supporting its applicability in hospitals with less mature ASP structures.

In addition to comparing process indicators of the ASP, the study also included the evaluation of outcome indicators, such as the incidence of CDI, mortality, and readmission rates. In the intervention cohort, an audit-and-feedback process was conducted for all antibiotic prescriptions. The cohorts represent two distinct time periods, ensuring the absence of contamination between the groups. The high acceptance rate of the recommendations (>95%) indicates that nearly all patients in the intervention cohort received treatment in accordance with the ASP.

Our study presents some limitations. This is a single-center quasi-experimental study comparing two patient cohorts at different times during the pandemic, with the inherent limitations of such studies. In addition to the direct effect of the intervention, other unexplored factors may have influenced prescribing behavior. Although multivariate adjustment was performed using multiple indicators of baseline severity, the quasi-experimental before-and-after design cannot eliminate the risk of residual confounding. Given that the ASP cohort exhibited a higher clinical severity at baseline, it is possible that unmeasured differences between periods could partly influence the observed associations. However, the results across adjusted models support the robustness of the intervention effect. Although most multivariable models met the recommended events-per-variable ratio (Vittinghoff and McCulloch, 2007), the model assessing inappropriate antibiotic use in superinfection included a limited number of events. Therefore, these results should be interpreted cautiously, and residual overfitting cannot be completely excluded. In addition, the results of the sensitivity analysis, conducted to address potential residual confounding arising from differences in baseline severity, showed consistent findings after stratification by age and FEN-COVID phenotype. The results remained consistent across strata, except for underrepresented subgroups (phenotype A and antibiotic prescribing for coinfection in patients younger than 65 years). Additionally, the Hawthorne effect may have altered prescription patterns. Antibiotic treatment audits included a subjective component, conducted retrospectively in the pre-intervention cohort and prospectively in the intervention cohort. Given the absence of a gold standard for initiating antibiotic therapy in COVID-19 patients, prescriptions were assessed using clinical, analytical, microbiological, and radiological criteria, alongside local hospital guidelines. The pre-intervention cohort was evaluated retrospectively, whereas the ASP cohort was assessed prospectively. Although both periods were reviewed by the same ASP team using standardized criteria and a paired-review approach, some variability related to the different modes of assessment cannot be fully excluded. The economic impact of these interventions remains unassessed.

As the pandemic progressed and data on rates of coinfection and superinfection associated with COVID-19 became available, the proportion of patients receiving empirical antibacterial therapy decreased (Sili et al., 2024). The evolving phases of the pandemic likely influenced antibiotic prescribing by altering diagnostic capacity, clinical experience, and treatment protocols. Because the pre-ASP period already encompassed both early and intermediate phases, part of the natural decline in empirical antibiotic use was captured in the baseline data. Despite the higher baseline severity in the ASP cohort, inappropriate prescribing still decreased significantly after the intervention, suggesting that the observed effect is not solely explained by temporal trends. Nevertheless, some residual confounding related to unmeasured pandemic-phase factors cannot be fully excluded.

In conclusion, despite the challenges posed by the COVID-19 pandemic, our hospital, building on its long-standing antimicrobial stewardship tradition, implemented a targeted ASP for hospitalized COVID-19 patients during the third and fourth waves. This three-step, non-restrictive strategy, based on pharmacist-led pre-evaluation and follow-up combined with audit and feedback from infectious disease specialists, was associated with a reduction in antibiotic consumption (DOT/1,000 patient-days), overall antibiotic use, inappropriate prescribing, and treatment duration, while maintaining patient safety. These findings suggest that the ASP likely contributed to optimizing antibiotic therapy in this setting. Moreover, identifying factors associated with antibiotic prescription and inappropriate use may help inform the development of targeted ASP strategies in similar clinical contexts.

## Data Availability

The raw data supporting the conclusions of this article will be made available by the authors, without undue reservation.
