# Supplementary material for: Effectiveness and safety of a prospective audit and feedback-based antimicrobial stewardship program in hospitalized COVID-19 patients: a quasi-experimental before-and-after study
Source: Front Pharmacol. 2026 Mar 20;17:1662195. doi: 10.3389/fphar.2026.1662195 (PMC13047313; doi:10.3389/fphar.2026.1662195)
Supplement: Supplementary file 1 [file Table1.docx]

Supplementary Table 1. Univariate and multivariate analysis of risk factors associated to antibiotic prescription for coinfection.

| Variable | Antibiotic for coinfection n (%)  No Yes | | Crude OR (IC 95%) | P value | Adjusted OR (IC 95%) | P value |
| --- | --- | --- | --- | --- | --- | --- |
| Demographic characteristics | | | | | | |
| Male sex | 636 (57,8) | 115 (60,8) | 1,13 (0,83-1,56) | 0,435 | - | - |
| Age ≥ 80 years | 195 (17,7) | 53 (28,0) | 1,81 (1,27-2,58) | 0,001 | - | - |
| Baseline characteristics | | | | | | |
| Comorbidities | | | | | | |
| Pulmonary chronic disease | 169 (15,4) | 47 (24,9) | 1,82 (1,26-2,64) | 0,001 | 1,64 (1,09-2,46) | 0,017 |
| Obesity | 210 (19,1) | 31 (16,4) | 0,83 (0,55-1,26) | 0,381 | - | - |
| Heart failure/ ischemic heart disease | 297 (27) | 66 (34,9) | 1,45 (1,05-2,01) | 0,025 | - | - |
| Arterial hypertension | 568 (51,6) | 109 (57,7) | 1,28 (0,93-1,74) | 0,125 | - | - |
| Asthma | 87 (7,9) | 12 (6,2) | 0,79 (0,42-1,47) | 0,457 | - | - |
| Chronic kidney disease | 68 (6,2) | 21 (11,1) | 1,90 (1,13- 3,18) | 0,014 | - | - |
| Cirrhosis | 13 (1,2) | 1 (0,5) | 0,45 (0,06-3,42) | 0,424 | - | - |
| Solid neoplasm | 60 (5,5) | 25 (13,5) | 2,64 (1,61-4,33) | <0,001 | 2,22 (1,28-3,86) | 0,005 |
| Hematologic malignancy | 13 (1,2) | 6 (3,2) | 2,74 (1,03-7,3) | 0,036 | - | - |
| Diabetes mellitus | 263 (23,9) | 57 (30,2) | 1,37 (0,98-1,93) | 0,066 | - | - |
| Dementia | 65 (5,9) | 23 (12,2) | 2,21 (1,33-3,65) | 0,002 | 2,17 (1,25-3,76) | 0,006 |
| Baseline therapy | | | | | | |
| Corticosteroids | 40 (3,6) | 11 (5,8) | 1,64 (0,83-3,25) | 0,155 | - | - |
| Chemotherapy | 10 (0,9) | 6 (3,2) | 3,57 (1,28-9,95) | 0,009 | - | - |
| Biologic agents | 2 (0,2) | 3 (1,6) | 8,86 (1,47-53,35) | 0,004 | 8,22 (1,17-57,87) | 0,034 |
| Laboratory parameters on admission | | | | | | |
| Leukocytes ≥ 14000 /μL3 | 34 (3,1) | 32 (16,9) | 6,39 (3,83-10,65) | <0,001 | 5,46 (3,13-9,52) | <0,001 |
| Lymphocytes <1000 /μL3 | 545 (49,5) | 127 (67,2) | 2,09 (1,51-2,89) | <0,001 | 1,98 (1,39-2,82) | <0,001 |
| CRP ≥100 mg/L | 352 (32) | 112 (59,3) | 3,09 (2,25-4,24) | <0,001 | 2,95 (2,10-4,15) | <0,001 |
| Saturation 02 < 88% | 145 (13,2) | 46 (24,3) | 2,12 (1,46-3,08) | <0,001 | - | - |
| ASP | | | | | | |
| ASP intervention | 637 (57,9) | 91 (48,1) | 0,68 (0,50-0,92) | 0,012 | 0,47 (0,33-0,66) | <0,001 |

Supplementary Table 2. Univariate and multivariate analysis of risk factors associated to antibiotic prescription for superinfection.

| Variable | Antibiotic for superinfection n (%)  No Yes | | Crude OR (IC 95%) | P value | Adjusted OR (IC 95%) | P value |
| --- | --- | --- | --- | --- | --- | --- |
| Demographic characteristics | | | | | | |
| Male sex | 627 (58,0) | 124 (59,6) | 1,07 (0,79-1,45) | 0,666 | - | - |
| Age ≥ 65 years | 534 (49,4) | 142 (68,3) | 2,20 (1,61-3,02) | <0,001 | 1,66 (1,18-2,34) | 0,004 |
| Baseline characteristics | | | | | | |
| Comorbidities | | | | | | |
| Pulmonary chronic disease | 162 (15,0) | 54 (26,0) | 1,99 (1,40-2,83) | <0,001 | 1,49 (1,02-2,16) | 0,039 |
| Obesity | 194 (17,9) | 47 (22,6) | 1,34 (0,93-1,91) | 0,115 | - | - |
| Heart failure/ ischemic heart disease | 281 (26,0) | 82 (39,4) | 1,85 (1,36-2,53) | <0,001 | - | - |
| Arterial hypertension | 543 (50,2) | 134 (64,4) | 1,80 (1,32-2,44) | <0,001 | - | - |
| Asthma | 81 (7,5) | 18 (8,7) | 1,17 (0,69-2,0) | 0,565 | - | - |
| Chronic kidney disease | 60 (5,6) | 29 (13,9) | 2,76 (1,72-4,42) | <0,001 | 2,01 (1,22-3,34) | 0,007 |
| Cirrhosis | 12 (1,1) | 2 (1,0) | 0,87 (0,19-3,89) | 0,850 | - | - |
| Solid neoplasm | 56 (5,2) | 29 (13,9) | 2,97 (1,84-4,77) | <0,001 | 2,48 (1,50-4,09) | <0,001 |
| Hematologic malignancy | 15 (1,4) | 4 (1,9) | 1,39 (0,46-4,24) | 0,557 | - | - |
| Diabetes mellitus | 254 (23,5) | 66 (31,7) | 1,51 (1,09-2,09) | 0,012 | - | - |
| Dementia | 56 (5,2) | 32 (15,4) | 3,33 (2,10-5,29) | <0,001 | 2,82 (1,71-4,63) | <0,001 |
| Baseline therapy | | | | | | |
| Corticosteroids | 39 (3,6) | 12 (5,8) | 1,64 (0,84-3,18) | 0,143 | - | - |
| Chemotherapy | 13 (1,2) | 3 (1,4) | 1,20 (0,34-4,26) | 0,775 | - | - |
| Biologic agents | 3 (0,3) | 2 (1) | 3,49 (0,58-21,01) | 0,146 | - | - |
| COVID-19 therapy | | | | | | |
| Remdesivir | 53 (4,9) | 11 (5,3) | 1,08 (0,56-2,11) | 0,815 | - | - |
| Corticosteroids | 891 (82,4) | 196 (94,2) | 3,48 (1,91-6,40) | <0,001 | 3,35 (1,79-6,24) | <0,001 |
| Tocilizumab | 118 (10,9) | 34 (16,3) | 1,60 (1,05-2,41) | 0,026 | 1,77 (1,13-2,76) | 0,012 |
| ASP | | | | | | |
| ASP intervention | 610 (56,4) | 118 (56,7) | 1,01 (0,75-1,37) | 0,936 | 0,72 (0,52-1,00) | 0,052 |

Supplementary Table 3. Univariate and multivariate analysis of risk factors associated to inappropriate antibiotic prescription for coinfection.

| Variable | Inappropriate antibiotic for coinfection n (%)  No Yes | | Crude OR (IC 95%) | P value | Adjusted OR (IC 95%) | | P value |
| --- | --- | --- | --- | --- | --- | --- | --- |
| Demographic characteristics | | | | | | | |
| Male sex | 704 (58,6) | 47 (54) | 0,83 (0,54-1,29) | 0,406 | | - | - |
| Age ≥ 65 years | 624 (51,9) | 52 (59,8) | 1,38 (0,88-2,14) | 0,156 | | - | - |
| Baseline characteristics | | | | | | | |
| Comorbidities | | | | | | | |
| Pulmonary chronic disease | 200 (16,6) | 16 (18,4) | 1,13 (0,64-1,98) | 0,673 | | - | - |
| Obesity | 227 (18,9) | 14 (16,1) | 0,82 (0,46-1,49) | 0,519 | | - | - |
| Heart failure/ ischemic heart disease | 333 (27,7) | 30 (34,5) | 1,37 (0,87-2,18) | 0,175 | | - | - |
| Arterial hypertension | 631 (52,5) | 46 (52,9) | 1,02 (0,66-1,57) | 0,946 | | - | - |
| Asthma | 91 (7,6) | 8 (9,2) | 1,24 (0,58-2,64) | 0,583 | | - | - |
| Chronic kidney disease | 82 (6,8) | 7 (8) | 1,20 (0,54-2,67) | 0,664 | | - | - |
| Cirrhosis | 13 (1,1) | 1 (1,1) | 1,06 (0,14-8,23) | 0,953 | | - | - |
| Solid neoplasm | 72 (6,0) | 13 (14,9) | 2,76 (1,46-5,21) | 0,001 | | 2,96 (1,52-5,77) | 0,001 |
| Hematologic malignancy | 17 (1,4) | 2 (2,3) | 1,64 (0,37-7,22) | 0,509 | | - | - |
| Diabetes mellitus | 293 (24,4) | 27 (31) | 1,40 (0,87-2,24) | 0,165 | | - | - |
| Dementia | 80 (6,7) | 8 (9,2) | 1,42 (0,66-3,04) | 0,364 | | - | - |
| Baseline therapy | | | | | | | |
| Corticosteroids | 47 (3,9) | 4 (4,6) | 1,18 (0,42-3,37) | 0,751 | | - | - |
| Chemotherapy | 15 (1,2) | 1 (1,1) | 0,92 (0,12-7,05) | 0,936 | | - | - |
| Biologic agents | 4 (0,3) | 1 (1,1) | 3,48 (0,39-31,5) | 0,237 | | - | - |
| Laboratory parameters on admission | | | | | | | |
| Leukocytes ≥ 14000 /μL3 | 57 (4,7) | 9 (10,3) | 2,32 (1,11-4,86) | 0,022 | | - | - |
| Lymphocytes <1200 /μL3 | 423 (35,2) | 23 (26,4) | 0,66 (0,41-1,08) | 0,097 | | - | - |
| CRP ≥100 mg/L | 413 (34,4) | 51 (58,6) | 2,71 (1,74-4,22) | <0,001 | | 2,71 (1,69-4,34) | <0,001 |
| Saturation 02 < 92% | 456 (37,9) | 44 (50,6) | 1,67 (1,08-2,59) | 0,019 | | 1,65 (1,03-2,64) | 0,038 |
| ASP | | | | | | | |
| ASP intervention | 699 (58,2) | 29 (33,3) | 0,36 (0,23-0,57) | <0,001 | | 0,27 (0,17-0,44) | <0,001 |

Supplementary Table 4. Univariate and multivariate analysis of risk factors associated to inappropriate antibiotic prescription for superinfection.

| Variable | Inappropriate antibiotic for superinfection n (%)  No Yes | | Crude OR (IC 95%) | P value | Adjusted OR (IC 95%) | P value |
| --- | --- | --- | --- | --- | --- | --- |
| Demographic characteristics | | | | | | |
| Male sex | 710 (58,3) | 41 (57,7) | 0,98 (0,60-1,59) | 0,928 | - | - |
| Age ≥ 60 years | 730 (59,9) | 54 (76,1) | 2,12 (1,22-3,71) | 0,007 | 1,86 (1,03-3,35) | 0,041 |
| Baseline characteristics | | | | | | |
| Comorbidities | | | | | | |
| Pulmonary chronic disease | 196 (16,1) | 20 (28,2) | 2,05 (1,19-3,51) | 0,008 | - | - |
| Obesity | 222 (18,2) | 19 (26,8) | 1,64 (0,95-2,83) | 0,073 | 1,86 (1,05-3,29) | 0,036 |
| Heart failure/ ischemic heart disease | 334 (27,4) | 29 (40,8) | 1,83 (1,12-2,98) | 0,015 | - | - |
| Arterial hypertension | 633 (52) | 44 (62) | 1,51 (0,92-2,47) | 0,101 | - | - |
| Asthma | 92 (7,6) | 7 (9,9) | 1,34 (0,60-3,00) | 0,478 | - | - |
| Chronic kidney disease | 80 (6,6) | 9 (12,7) | 2,07 (0,99-4,31) | 0,048 | - | - |
| Cirrhosis | 14 (1,1) | - |  | 0,364 | - | - |
| Solid neoplasm | 74 (6,1) | 11 (15,5) | 2,83 (1,43-5,62) | 0,002 | 2,88 (1,41-5,87) | 0,005 |
| Hematologic malignancy | 18 (1,5) | 1 (1,4) | 0,96 (0,13-7,24) | 0,962 |  |  |
| Diabetes mellitus | 299 (24,5) | 21 (29,6) | 1,29 (0,76-2,19) | 0,340 |  |  |
| Dementia | 77 (6,3) | 11 (15,5) | 2,72 (1,37-5,38) | 0,003 | 3,00 (1,45-6,23) | 0,003 |
| Baseline therapy | | | | | | |
| Corticosteroids | 44 (3,6) | 7 (9,9) | 2,92 (1,27-6,74) | 0,009 | 3,44 (1,44-8,22) | 0,005 |
| Chemotherapy | 15 (1,2) | 1 (1,4) | 1,15 (0,15-8,80) | 0,896 | - | - |
| Biologic agents | 5 (0,4) | - |  | 0,589 | - | - |
| COVID-19 therapy | | | | | | |
| Remdesivir | 62 (5,1) | 2 (2,8) | 0,54 (0,13-2,26) | 0,391 | - | - |
| Corticosteroids | 1020 (83,7) | 67 (94,4) | 3,25 (1,17-9,02) | 0,017 | - | - |
| Tocilizumab | 135 (11,1) | 17 (23,9) | 2,53 (1,42-4,48) | 0,001 | 3,43 (1,84-6,39) | <0,001 |
| ASP | | | | | | |
| ASP intervention | 690 (56,7) | 38 (53,5) | 0,88 (0,55-1,42) | 0,605 | 0,66 (0,40-1,11) | 0,116 |

Supplementary Table 5. Sensitivity Analysis. Outcome variables and age > 65 years

| Age | Outcome | Pre- ASP | | ASP | P value |
| --- | --- | --- | --- | --- | --- |
| ≤ 65 years | Antibiotics for bacterial coinfection (all causes) | 14 (13.4) | 33 (9.6) | | 0.134 |
|  | Antibiotics for bacterial superinfection (all causes) | 31 (10.4) | 44 (12.9) | | 0.334 |
|  | Incorrect use of antibiotics for bacterial coinfection (all causes) | 27 (9.1) | 8 (2.3) | | <0.001 |
|  | Incorrect use of antibiotics for bacterial superinfection (all causes) | 11 (3.7) | 16 (4.7) | | 0.530 |
| > 65 years | Antibiotics for bacterial coinfection (all causes) | 58 (22.1) | 58 (15.0) | | 0.022 |
|  | Antibiotics for bacterial superinfection (all causes) | 59 (22.4) | 74 (19.2) | | 0.312 |
|  | Incorrect use of antibiotics for bacterial coinfection (all causes) | 31 (11.8) | 21 (5.4) | | 0.003 |
|  | Incorrect use of antibiotics for bacterial superinfection (all causes) | 22 (8.4) | 22 (5.7) | | 0.185 |

Supplementary Table 6. Sensitivity Analysis. Outcome variables and COVID-19 phenotype.

| FEN-COVID | Outcome | Pre- ASP | | ASP | P value |
| --- | --- | --- | --- | --- | --- |
| Phenotype A | Antibiotics for bacterial coinfection (all causes) | 14 (13.5) | 4 (13.3) | | 0.986 |
|  | Antibiotics for bacterial superinfection (all causes) | 13 (12.5) | 4 (13.3) | | 0.904 |
|  | Incorrect use of antibiotics for bacterial coinfection (all causes) | 8 (7.7) | 1 (3.3) | | 0.401 |
|  | Incorrect use of antibiotics for bacterial superinfection (all causes) | 5 (4.8) | 0 (0.0) | | 0.221 |
| Phenotype B-C | Antibiotics for bacterial coinfection (all causes) | 84 (18.4) | 87 (12.5) | | 0.006 |
|  | Antibiotics for bacterial superinfection (all causes) | 77 (16.8) | 114 (16.3) | | 0.817 |
|  | Incorrect use of antibiotics for bacterial coinfection (all causes) | 50 (10.9) | 28 (4.0) | | <0.001 |
|  | Incorrect use of antibiotics for bacterial superinfection (all causes) | 28 (6.1) | 38 (5.5) | | 0.629 |
